# Insulator Surface Defect Detection Method Based on Graph Feature Diffusion Distillation

**DOI:** 10.3390/jimaging11060190

**Published:** 2025-06-10

**Authors:** Shucai Li, Na Zhang, Gang Yang, Yannong Hou, Xingzhong Zhang

**Affiliations:** 1 State Grid Shanxi Electric Power Company Lvliang Power Supply Company, Lvliang 033000, China; lishucai@sx.sgcc.com.cn; 2 State Grid Shanxi Electric Power Company Electric Power Research Institute, Taiyuan 030001, China; zhangna@sx.sgcc.com.cn; 3 State Grid Shanxi Electric Power Company Ultra High Voltage Substation Branch, Taiyuan 030021, China; cgyhouyannong@sx.csgcc.com.cn; 4 School of Software, Taiyuan University of Technology, Taiyuan 030001, China; zhangn25@126.com

**Keywords:** defect detection, unsupervised learning, teacher-student networks, graph features, knowledge distillation

## Abstract

Aiming at the difficulties of scarcity of defect samples on the surface of power insulators, irregular morphology and insufficient pixel-level localization accuracy, this paper proposes a defect detection method based on graph feature diffusion distillation named GFDD. The feature bias problem is alleviated by constructing a dual-division teachers architecture with graph feature consistency constraints, while the cross-layer feature fusion module is utilized to dynamically aggregate multi-scale information to reduce redundancy; the diffusion distillation mechanism is designed to break through the traditional single-layer feature transfer limitation, and the global context modeling capability is enhanced by fusing deep semantics and shallow details through channel attention. In the self-built dataset, GFDD achieves 96.6% Pi.AUROC, 97.7% Im.AUROC and 95.1% F1-score, which is 2.4–3.2% higher than the existing optimal methods; it maintains excellent generalization and robustness in multiple public dataset tests. The method provides a high-precision solution for automated inspection of insulator surface defect and has certain engineering value.

## 1. Introduction

Insulators, as an important component of transmission lines, can effectively isolate wires and towers, prevent the current from entering the ground through the tower, avoid ground faults on the line, and guarantee the transmission of electric energy according to the established path. However, in the complex external environment, its surface is susceptible to breakage, self-explosion, flashover and other faults due to the influence of the natural environment [1], which may lead to large-scale power outage and cause serious economic losses if not detected and taken measures in time. Therefore, improving the efficiency and accuracy of insulator surface defect detection can detect and repair potential defect problems in advance, thus reducing the risk of failure and improving the reliability and safety of the power system.

At this stage, insulator surface defect detection is mainly categorized into supervised learning-based methods and unsupervised learning-based methods. Supervised learning-based methods [2,3,4] utilize sample data with marker information for learning, thus realizing the detection of abnormal samples. For example, Tomaszewski et al. [5] combined frequency-domain feature extraction with integrated learning through an innovative method to achieve an F1 score of 0.932 in power insulator fault detection, but its detection accuracy is highly dependent on sample color intensity profiles, and its generalization ability in real-world scenarios is yet to be verified. Zhao et al. [6] used a model that fused global context and local spatial information The dual-branch structure of YOLOv7 enhances the ability to obtain key information, and at the same time combines with data enhancement techniques to improve the generalization ability of the model in different environments, and its MAP improves by 8.9% compared to YOLOv7, but its detection efficiency needs to be improved. Xu et al. [7] designed the MAP-CA attention mechanism, which effectively fuses the global and local information, and optimized the structure of the YOLOv8 network, and its optimal f1 score reaches 0.98. Wang et al. [8] improved the YOLOv9 algorithm by improving the GAM attention mechanism to improve the accuracy of insulator fault detection, and its average detection accuracy reaches 0.966. However, the algorithm can only detect labeled defect types, which leads to the algorithm having certain limitations in practical applications. The above methods improve and optimize the insulator defect detection task from different perspectives, but these methods rely on large-scale labeled defect samples, which cannot achieve pixel-level defect detection, and the detection capability decreases dramatically in the face of unknown defect types, which makes it difficult to be applied in practice.

The above methods improve and optimize the insulator defect detection task from different perspectives, but these methods rely on large-scale labeled defect samples and cannot achieve pixel-level defect detection as well as facing unknown defect types, the detection capability decreases dramatically, making it difficult to meet the complex and varied defect characterization in real-world scenarios.

With the rapid development of deep learning techniques, related methods based on unsupervised learning are also developing rapidly. Unlike supervised learning, unsupervised methods do not need to rely on labeled defective data. Instead, they learn the intrinsic distribution of normal samples through self-supervised reconstruction or density estimation, and achieve anomaly detection through hidden space mapping or reconstruction error. For example, Liu et al. [9] proposed a density-based spatial clustering method, which realizes the detection of defective regions by setting different thresholds according to the clustering results. Wang Dao lei et al. [10] used U-Net for image reconstruction to extract image features using jump connection multi-scale, so that the reconstructed image contains more details of the real image, and the quality of the reconstructed image is improved from the image and potential space, but it is difficult for this method to realize the detection of defects in multiple categories. The above image reconstruction-based method locates defective regions in the image based on the reconstruction error, but due to the strong generalization ability of convolutional neural networks, defective regions may also be correctly reconstructed in the inference stage, making it difficult to effectively distinguish defective regions from normal regions.

Feature embedding-based methods show better performance in many scenarios, and commonly used feature embedding-based anomaly detection can be classified as memory banks, feature mapping and knowledge distillation [11]. Cohen et al. [12] identify an image as normal or abnormal based on whether the K-nearest neighbors (KNN) distance is greater than a threshold by finding the pixel-level correspondence between the test image and the nearest neighboring normal image. However, the KNN method ignores the local density bias due to the feature local density bias due to uneven data distribution in the space, which affects the detection accuracy. Roth et al. [13] address the limitations of traditional methods relying solely on final-layer features from pre-trained networks by instead leveraging intermediate-layer representations for normal samples. This approach preserves fine-grained spatial details and enhances contextual awareness of defects. However, such methods often introduce intra-layer feature redundancy and single-scale bias during memory bank construction, leading to suboptimal storage efficiency and incomplete defect characterization. To mitigate these issues, recent studies have integrated knowledge distillation frameworks. For example, Tong et al. [14] proposed a two-stage training algorithm based on reverse knowledge distillation, which uses a memory bank to store representative prototypical features and suppresses the decoding of anomalous features by the student network to ensure efficient model reconstruction. TIEN et al. [15], inspired by the reverse distillation framework, proposed combining it with multitasking learning to achieve compact feature representation and anomaly mitigation. However, the existing methods transitionally rely on a single feature in the teacher network, and the pre-trained teacher network often suffers from knowledge bias.

To address the limitations of existing teacher-student frameworks in feature representation diversity and knowledge bias, this study proposes a defect detection algorithm based on graph feature diffusion distillation. The method employs dual-teacher encoders—one pre-trained and one non-pre-trained—to capture complementary features, introducing a graph feature consistency module to mitigate pre-training knowledge bias and enhance target-domain feature representation. An inter-layer feature fusion module dynamically combines multi-layer features from dual teachers to suppress redundancy and retain critical information, while a diffusion distillation mechanism guided by channel attention transfers deep semantic information to shallow layers, breaking through traditional single-layer feature alignment constraints and enhancing the student network’s understanding of global contextual information for robust pixel-level defect detection across scales.

## 2. Related Work

### 2.1. Image Reconstruction Approaches

Image reconstruction-based methods, such as autoencoders [16,17] and generative adversarial networks (GANs) [18,19], train models to reconstruct input images by minimizing pixel-wise reconstruction errors. The core premise is that normal images can be reconstructed with minimal error, enabling defect localization by identifying regions with high reconstruction error. However, neural networks’ powerful learning ability often leads to the effective reconstruction of defective regions as well, which reduces the accuracy of defect detection based on this error. To improve reconstruction quality, Lee [20] employed a ViT-based model that uses transposed convolutional layers to reconstruct same-size images from feature maps, then applied the reconstruction error for defect identification. Similarly, De [21] introduced multishape and multiscale masking of input image patches and incorporated a masking component into ViT’s self-attention module. This forces each patch to rely solely on contextual information while ignoring its own potentially anomalous data. Nevertheless, during inference, these reconstruction models remain susceptible to distortion from defective regions, preventing pixel-level defect localization. You [22] proposed a neighborhood mask attention mechanism to achieve fine-grained anomaly localization via local feature alignment, though the method’s generalization across multi-category defect detection requires further improvement. Unlike these approaches, the method presented in this paper avoids image reconstruction entirely and instead performs end-to-end defect localization.

### 2.2. Feature Embedding Approaches

Feature embedding-based methods typically utilize a network pre-trained on ImageNet to extract features from input images. Defective regions are then located by calculating feature distances between test samples and normal samples to derive anomaly scores. Zheng [23] designed a two-stage coarse-to-fine feature alignment network to learn robust feature distributions for normal images. Defard [24] proposed the Padim model, which extracts patch features using a pre-trained CNN and models normal samples with a multivariate Gaussian distribution. Building on this, WAN [25] introduced the PFM method to enhance model robustness through hierarchical feature matching. Embedding-based methods are widely adopted due to their simplicity and effectiveness. However, their performance largely depends on the robustness of features stored in the feature memory bank and requires complex feature matching during inference, significantly limiting model speed.

To address these limitations, some researchers have incorporated knowledge distillation. Liu Tao [26] used teacher-generated pseudo-labels to train a student network, achieving 90.13% accuracy on tile category detection while enabling real-time performance through model compression. Wang [27] distilled teacher knowledge into a single student network using hierarchical feature matching, enabling multi-scale learning to reduce information loss and improve segmentation accuracy. Similarly, Salehi [28] proposed a multi-resolution knowledge distillation framework to locate anomalies through multi-layer feature differences in teacher-student networks. Zolfaghari [29] optimized feature representations using a teacher-student feature pyramid matching strategy with fine-tuning to improve detection accuracy for irregular defects. Nevertheless, these approaches rely on pre-trained models to extract generalized features, which often fail to capture fine-grained local characteristics or domain-specific patterns of industrial defects, leading to missed detections or false positives.

## 3. Methods

### 3.1. Overall Framework

**Figure 1:**
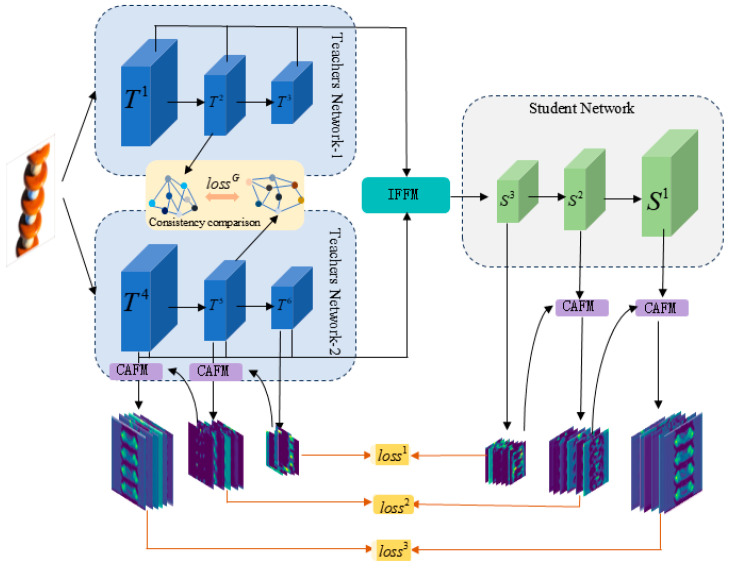
The overall architecture of the proposed method has three key components: Graph Feature Comparison Module (GFCM), Interlayer Feature Fusion Module (IFFM), Channel Attention Fusion Module (CAFM), indicated by yellow, green and purple color, respectively.

The general framework of the proposed method is shown in Figure 1.This paper adopts dual-teacher encoders to extract abstract features from the image, utilize the graph feature consistency module (Section 3.2) to mitigate the feature bias among encoders, design the interlayer feature module (Section 3.3) to efficiently integrate different layers of features from dual-teachers, and provide the decoder (the network of students) with more complete semantic features. In the feature reconstruction stage, the synergy of channel attention module (Section 3.4) and diffusion distillation approach (Section 3.5) is used to achieve multi-layer feature transfer from deep to shallow, and finally pixel-level defect detection is achieved through multi-scale (Section 3.6) feature differences.

### 3.2. Graph Feature Comparison Module

**Figure 2:**
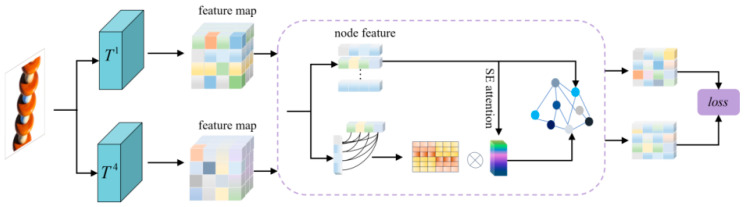
Structure of the graph feature comparison module. The dashed box shows the graph structure construction process where the upper branches are the nodes and the lower branches are the edges.

In traditional teacher-student frameworks, relying solely on pre-trained teacher networks often introduces cross-domain feature bias, particularly in cross-scene industrial applications (e.g., insulator defect detection). Here, single-teacher models pre-trained on ImageNet [30] struggle to adapt to diverse industrial defect patterns (e.g., complex surface cracks, discharge traces), as their feature encoders are constrained by ImageNet’s natural-image distribution, leading to degraded cross-scene generalization. To tackle this, we introduce a dual-teacher collaborative architecture, where the graph feature comparison module enables csemantic alignment by modeling long-range feature dependencies via graph convolution. This mechanism provides the dual-teacher ensemble with a global contextual perspective, mitigating pre-training domain bias and enhancing their sensitivity to fine-grained insulator defects through structured feature interaction. As illustrated in Figure 2, the module employs a graph neural network to encode feature relationships across dual teachers, enabling joint optimization of local feature discriminability and global semantic consistency.

In defect detection tasks, industrial defects typically exhibit non-local distributions with multi-scale correlations. While it is difficult to establish irregular remote spatial dependencies with the fixed receptive fields of conventional CNNs, the graph structure can explicitly encode remote dependencies between features through the flexible definition of nodes and edges [31]. So this section designs the graph feature comparison module to mitigate the sample feature bias by using graph features to capture the remote dependencies of different channel dimensions. Figure 2 takes the shallow coding in dual-teacher encoder as an example, and the H×W feature matrix of each channel is spread into 1×N(N=H×W) vectors as graph nodes, forming a graph structure containing C nodes. To address the problem that the graph structure ignores the semantic correlation between the feature channels during the convolution process, this section introduces the channel attention, which captures the nonlinear correlation properties between the channels through dynamic weight learning to construct the edge information between the nodes. Specifically, for any two channel nodes, firstly, the spatial correlation between their channels is computed to obtain the co-activation strength $E_{i,j}^{raw}$ in the spatial dimension, as shown in Equation (1)

(1) $$E_{i,j}^{raw}=\frac{1}{H\times W}\sum_{h=1}^{H}\sum_{w=1}^{W}\psi_{i}(h,w)\cdot \psi_{j}(h,w)$$

where $\psi_{k}(,)\in {\mathbb{R}}^{H\times W}$ denotes the feature graph of channel *k*; $E_{i,j}^{raw}$ denotes the spatial co-activation strength of channel *i* and channel *j*;

Second, fusing the a priori knowledge of single-channel importance, the channel weight $S\in R^{C\times 1}$ output from SE Attention (Squeeze-and-Excitation Attention) [32] is introduced into the edge weight calculation, and the scalar weights $S_{i}$ and $S_{j}$ are connected as two-dimensional weights to generate the final edges through the learnable parameter matrix $W_{g}\in R^{3\times 1}$, which is calculated as follows:

(2) $$E_{i,j}=\mathrm{sigmod}(W_{g}\cdot \left[E_{i,j}^{raw},S_{i},S_{j}\right])$$

The topology optimization and spatial information propagation of the graph features at each level is performed by multilayer graph convolution operation [33], and the graph structure feature projection is restored to the original spatial scale with the help of inverse convolution and channel reorganization. In high-dimensional feature spaces, the Euclidean distance tends to converge to a similar distance between all samples due to the elevated feature dimensions, whereas cosine similarity passes through the similarity of vectors in terms of direction rather than absolute distance [34]. Therefore, we choose cosine similarity to construct multilayer feature comparisons, which is computed as shown below:

(3) $$\mathrm{sim}(a,b)=1-\frac{a^{T}\cdot b}{\left||a||||b|\right|}$$

Using Equation (3), the consistency of the features of each layer of the teacher network along the channel dimension is calculated layer by layer and the final consistency loss $Loss_{G}$ is obtained by using cumulative averaging, the calculation formula is shown in (4) and (5).

(4) $$L_{T1-T4}=\frac{1}{H\times W}\sum_{h=1}^{H}\sum_{w=1}^{W}\frac{1}{C}\sum_{i=1}^{C}\mathrm{sim}(V_{1}^{i}(h,w),V_{4}^{i}(h,w))$$

(5) $$Loss_{G}=\frac{1}{3}(L_{T1-T4}+L_{T2-T5}+L_{T3-T6})$$

where *C*, *H*, *W* denotes the number of channels, height and width of the feature map, respectively; $V_{j}^{i}(h,w)$ denotes the feature vector from channel i to channel *C* of the $T^{j}$ feature at the (h,w) position.

### 3.3. Interlayer Feature Fusion Module

**Figure 3:**
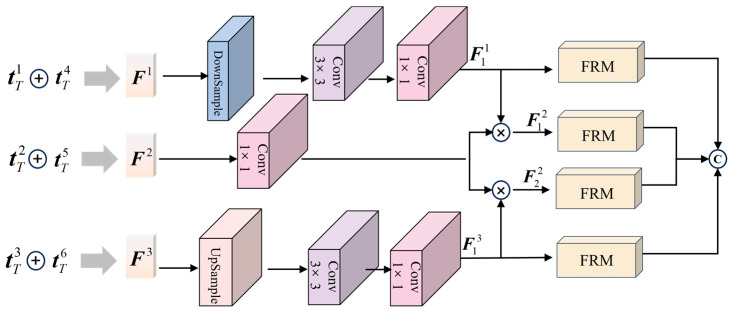
Structure of interlayer feature fusion module incorporating convolutional operations and feature reorganization module.

In traditional teacher—student network architectures, the last—layer deep features of the teacher network are often directly fed into the student network. However, this practice fails to fully exploit the semantic relationships among features across different layers, thereby compromising the reconstruction quality of the student network [35]. To alleviate this deficiency, we propose an interlayer feature fusion module and a feature reorganization module. As shown in Figure 3, the interlayer feature fusion module aims to integrate multi-scale features from a two-teacher network. This integration process includes using up-sampling and down-sampling to achieve alignment with intermediate layer features, and using convolution and multiplication operations to fuse features from different layers to capture rich semantic information. Then, the feature restructuring module further refines these fused features to achieve nonlinear restructuring of features using average pooling and maximum pooling of channel dimensions to provide high-quality inputs to the student network. This combination approach effectively solves the problem of underutilization of features in traditional structures.

Specifically, we first fused the features of each layer of the different teacher networks by concatenation to obtain feature maps of different sizes, i.e., shallow feature $F^{1}$, intermediate feature $F^{2}$, and deeper feature $F^{3}$. Since the dimensions of these features vary, to facilitate subsequent processing, we down-sample $F^{1}$ and up-sample $F^{3}$ to match the dimension of the intermediate feature $F^{2}$. Then, we apply 3 × 3 convolution followed by 1 × 1 convolution. The 3 × 3 convolution extracts local features, and the 1 × 1 convolution reduces the dimension, thus extracting the abstract features from the down-sampled shallow feature and the up-sampled deep feature, denoted as $F_{1}^{1}$ and $F_{1}^{3}$ respectively. These abstract features represent the semantically enhanced shallow and deep features. Next, we multiply the extracted shallow and deep abstract features with the intermediate-layer features. After that, we feed the fused features into the feature reorganization module (FRM). The FRM is designed to optimize the feature representation. We concatenate the processed features from each step to obtain the final output. This process effectively combines multi—scale features from different teacher networks, providing high—quality input features for subsequent processing. The calculation formula is shown below:

(6) $$F_{1}^{1}=Conv_{1\times 1}(Conv_{3\times 3}(DownSample(F^{1})))$$

(7) $$F_{1}^{3}=Conv_{1\times 1}(Conv_{3\times 3}(UpSample(F^{3})))$$

(8) $$F_{out}=cat(FRM(F_{1}^{1}),FRM(F_{1}^{2}),FRM(F_{2}^{2}),FRM(F_{1}^{3}))$$

**Figure 4:**
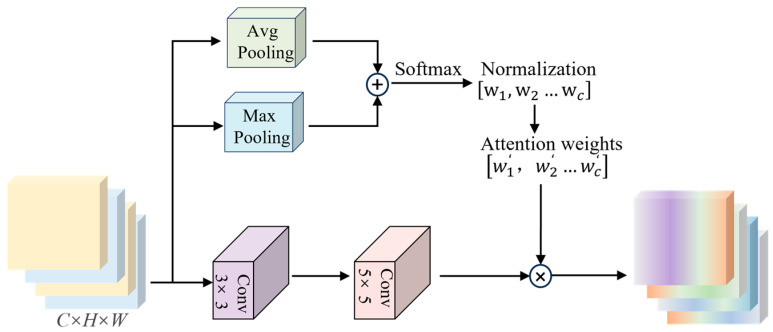
Structure of feature reorganization module with dual-pooling and convolutional operations for attention weight generation.

The proposed Feature Reorganization Module (FRM) introduces an innovative channel-wise attention mechanism through dual-channel pooling and dynamic weight allocation, effectively enhancing discriminative feature representation. As illustrated in Figure 4 the module employs a dual-branch architecture that simultaneously performs global max-pooling and average-pooling along the channel axis to capture complementary spatial-contextual information. These multi-scale feature descriptors are subsequently fused through element-wise summation and processed by an activation function to generate channel-wise attention coefficients. The normalized attention weights are then applied to recalibrate original features through channel-wise multiplication, achieving adaptive feature calibration. This novel design establishes cross-channel dependencies through nonlinear interactions between multi-granularity statistical features, enabling the network to dynamically emphasize informative channels while suppressing redundant ones. The architecture ensures computational efficiency while significantly improving model representational capacity through discriminative feature enhancement.

### 3.4. Channel Attention Fusion Module

**Figure 5:**
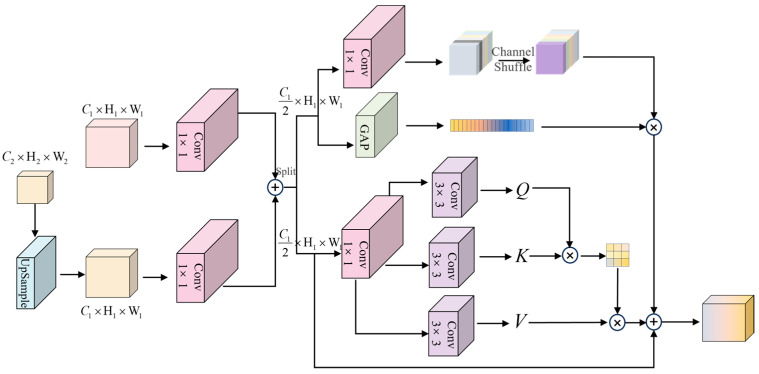
Channel Attention Fusion Module Structure: leveraging convolutional layers, channel shuffle and split for attention fusion.

In the traditional multi-layer distillation approach, feature transfer at different levels is performed independently, and the lack of explicit modeling of cross-level channel semantic associations leads to the inability of the student network to inherit cross-scale spatial dependencies implicit in the hierarchical knowledge of the teacher network. To address this limitation, we propose a cross-attention fusion module for the diffusion distillation path, which innovatively integrates channel attention with spatial self-attention mechanism to realize coherent reconstruction and discriminative enhancement of cross-level feature semantics by learning the association between channel features and global context information, providing the basis for the diffusion distillation approach in this paper, whose structure is shown in Figure 5.

In the channel attention fusion module, the features from different layers are unified into $C_{1}\times H_{1}\times W_{1}$ size by up-sampling operation, and then summed up by convolution operation. Then the features are divided into two parts of the same size by the Split operation, and the global and local feature enhancement is performed by the dual branching method. In the upper branch, the pooling operation on the channel dimension is used to obtain the intra-channel weights, and the channel shuffle is used to rearrange the channels, and finally the intra-channel weights are multiplied to obtain the inter-channel semantic correlation. In the lower branch Q, K, V are obtained by convolution operation and its global contextual features are obtained by using the self-attention mechanism [36], and finally the global features are element-wise summed with the local channel features to obtain the final fused features.

### 3.5. Diffusion Distillation

**Figure 6:**
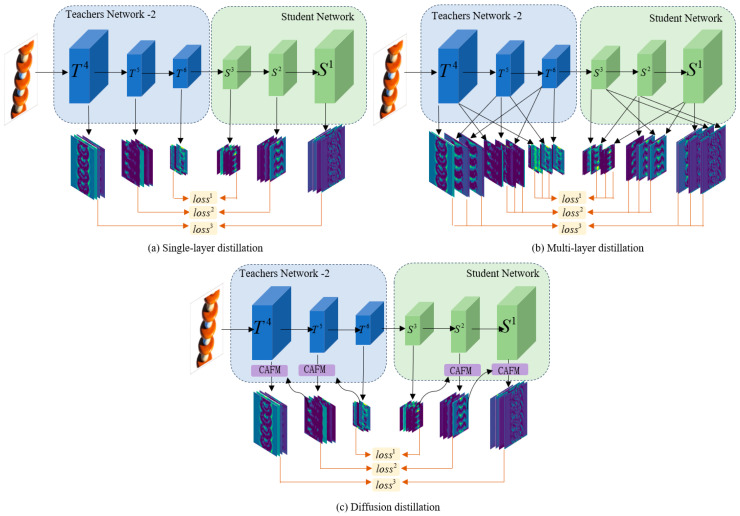
Distillation method (**a**) Single-layer distillation; (**b**) Multi-layer distillation; (**c**) Diffusion distillation.

In a typical teacher-student network, knowledge transfer between teacher-student networks is limited to the same feature layer. As shown in Figure 6a, this limited knowledge transfer often leads to a lack of necessary global contextual information in the student network, resulting in an inability to fully understand the acquired knowledge. To overcome this limitation, the multilayer distillation approach is then generated as shown in Figure 6b, where different layers of features are learned across scales through up-sampling and down-sampling operations, which makes full use of the semantic features at different levels, and cross-layer learning is performed between the deep teacher features and the shallow student features, which effectively expands the path of knowledge transfer between the student-teacher networks. However, this approach only performs feature alignment through sampling operations, ignoring the inherent semantic correlation between different feature channels, and the multiple complex distillation loss calculations greatly increase the burden of model training.

The traditional distillation is guided by knowledge only by the features between the corresponding layers, accumulating the losses of the different layers as a total loss function. In the case of vs. For example, the traditional method uses Equation (13) to learn only by between features of the corresponding scales:

(9) $$Loss^{t}=\frac{1}{H\times W}\sum_{h=1}^{H}\sum_{w=1}^{W}\frac{1}{C}\sum_{i=1}^{C}\mathrm{sim}(T_{i}^{6}(h,w),S_{i}^{3}(h,w))$$

The diffusion distillation method in this section, on the other hand, goes through the channel attention module to fuse the deep and shallow features, through which the deep features will be continuously diffused into the shallow features to enhance the global semantic relevance and form a multilevel complementary knowledge distillation path in the potential space. Its knowledge distillation can be represented as shown in Equation (14):

(10) $$Loss=\frac{1}{H\times W}\sum_{h=1}^{H}\sum_{w=1}^{W}\frac{1}{C}\sum_{i=1}^{C}sim(CAFM{\left(t_{T}^{5},t_{T}^{6}\right)}_{i},CAFM{\left(t_{S}^{2},t_{S}^{3}\right)}_{i})$$

where $t_{T}^{i}$ denotes the features extracted by the $T^{i}$ teacher encoding block; $t_{S}^{i}$ denotes the features extracted by the $S^{i}$ student decoding block; CAFM(a,b) denotes the feature map of *a* and *b* after fusion by the channel attention fusion module; and $CAFM{\left(,\right)}_{i}$ denotes the features from channel i to channel *C*.

### 3.6. Defect Detection

**Figure 7:**
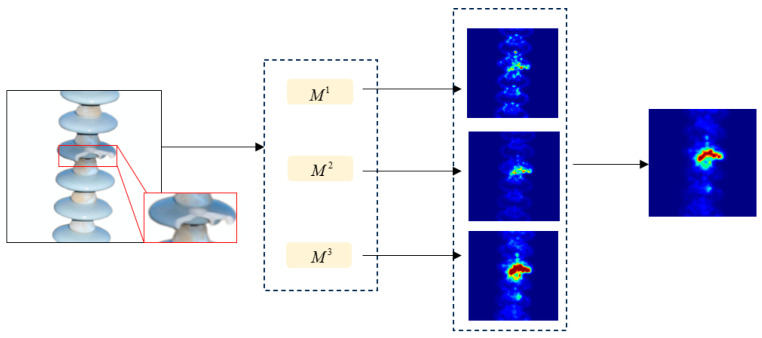
Defect Detection Process.

In the training phase, the student network focuses on learning the feature representation of the teacher network for normal samples. While facing unknown defects during testing, due to the lack of relevant knowledge, the student network will fail to reconstruct features in the defective region. Therefore, when the test sample contains unknown defects, there will be a feature difference between the reconstructed features of the student network and the extracted features of the teacher network, through which the defects can be detected. In order to quantify this difference, this section utilizes Equation (11) (with $M^{2}$ as the example) to generate a two-dimensional anomaly score map and obtains the final anomaly score map ***M*** through cumulative averaging. The anomaly score map is up-sample to the original image size, and the final anomaly score map is obtained by cumulative averaging, as shown in Figure 7.

(11) $$M^{2}=\frac{1}{C}\sum_{i=1}^{C}sim(CAFM{\left(t_{T}^{5},t_{T}^{6}\right)}_{i},CAFM{\left(t_{S}^{2},t_{S}^{3}\right)}_{i})$$

(12) $$M=\frac{1}{3}\sum_{k=1}^{3}\mathrm{Upsample}(M^{k})$$

where $M^{k}$ denotes the anomaly score map between the different layers corresponding to the teacher and student networks; Upsample(·) denotes the up-sampling.

## 4. Experimentation and Analysis

### 4.1. Datasets

**Figure 8:**
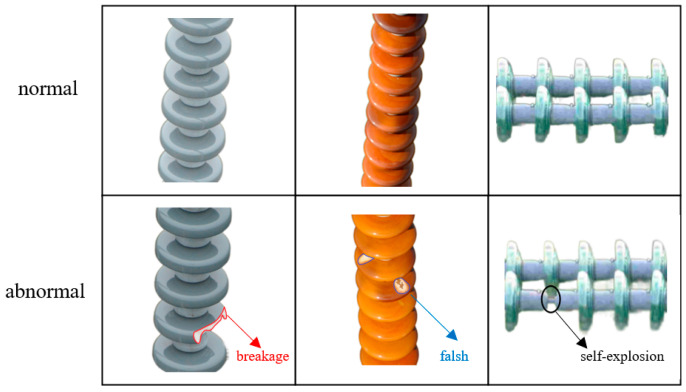
Example diagram of self-constructed dataset.

The experiments use the public industrial surface defect dataset MVtec AD [37], the public insulator dataset CID [38] with the self-constructed insulator dataset Wins, in which the self-constructed data images are obtained from the equipment resource management office of a national network. The self-constructed dataset contains a total of 1372 images, of which 1209 are normal sample images and 163 are abnormal sample images. The abnormal samples contain three common insulator defects, i.e., breakage, flash, and self-explosion, and each defect type is randomly stratified into a training set and a test set in the ratio of 8:2, with the specific details of the divisions as shown in Table 1. Examples of each type of defect samples are shown in Figure 8.The first column is breakage defects, i.e., physical breaks on the surface, which usually have irregular jagged edges (as shown in the red curved area); the second column is flashover defects, i.e., carbonized channels on the surface as a result of arc burning, which appear as dendrites or irregular traces (as shown in the blue curved area). The last column is the self-explosion defect, which often occurs in glass insulators and is characterized by local structural loss, as shown in the black curved area.

### 4.2. Experimental Platform and Parameter Setting

The experiments in this paper are based on NVIDIA GeForce RTX 3090 server for training and testing, the operating system is linux, CUDA11.3 version. The deep learning experiment platform is built based on Python3.8 and PyTorch 2.0.0. The input image size is 256 × 256 pixels; the batch size is set to 32, and the Adam optimizer is used with a learning rate of 0.001. The number of training rounds is set to 300 epochs, and the Early Stopping method is used to save the network parameters during the training process.

### 4.3. Evaluation Metrics

$F_{1}$ is the weighted sum average of precision and recall, when both precision and recall are high, F1-score is better, which represents better model detection. ROC curve is used in binary classification problems to measure the relationship between the model’s True Positive Rate (TPR) at different thresholds and the False Positive Rate (FPR). In this paper, we use F1-score with AUROC (Area Under the Receiver Operating Characteristic curve) for quantitative analysis, in which AUROC adopts Im.AUROC and Pi.AUROC. Im.AUROC is defined as the anomaly detection performance at the image level, reflecting the overall ability of the model to distinguish defective images from normal ones, and is computed as the area under the ROC curve of the predicted probability at the image level, while Pi.AUROC is defined as the defect localization performance at the pixel level, evaluating the accuracy of the model in identifying defective regions at the pixel level.

(13) $$Recall=\frac{TP}{TP+FN}$$

(14) $$Precision=\frac{TP}{TP+FP}$$

(15) $$F_{1}=2\times \frac{Precision\times Recall}{Precision+Recall}$$

where *FP* denotes the number of positive samples detected as negative; *TN* denotes the number of negative samples detected as positive; *TP* denotes the number of positive samples detected as positive; and *FN* denotes the number of negative samples detected as negative.

### 4.4. Comparison Experiment

This subsection provides a quantitative and observational analysis of the methods proposed in this chapter. To validate the effectiveness of the method, it is compared with the mainstream methods in the field of unsupervised defect detection through comparative experiments on the self-constructed dataset Wins and the publicly available dataset CID. To fully validate the effectiveness of the method in this paper, the experiments cover the following of comparison model: UniAD, Padim, PFM, MKD, ET-STPM.

#### 4.4.1. Comparison Experiment on Wins Dataset

The results of the self-built dataset defect detection are comprehensively presented in Table 2, Table 3 and Table 4, covering the three defect types as well as their average values. The experimental results show that the method in this paper has the optimal performance on self-built dataset defect detection, with its average Im.AUROC as high as 97.7%, which is better than the other models. In contrast, the MKD model performs poorly in defect detection, with an average Im.AUROC value of only 91%, which may be due to the single-teacher structure that causes the network to have a possible feature bias problem on the self-constructed dataset, which in turn increases the misdetection rate of defect detection. Similarly, UniAD performs poorly with its average Im.AUROC value of only 92.5%, the possible reason for this phenomenon is that there is a large intra-class variation in the normal samples of the self-constructed dataset, and the model is not able to learn the complex structured samples well.

**Figure 9:**
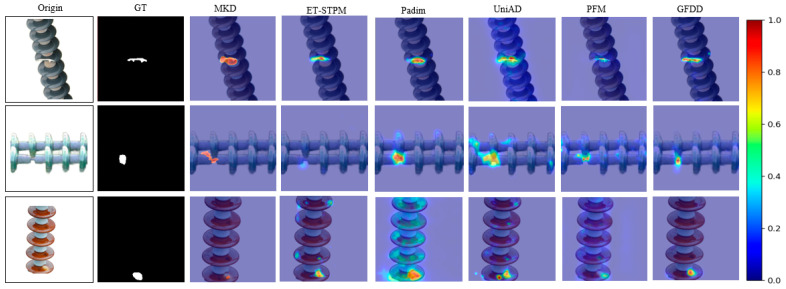
Results of wins heat map visualization.

For the segmentation of industrial surface defects, it is often necessary to pinpoint the exact location in the image where the defects appear. Therefore, pixel-level AUROC with F1 metrics is used for quantitative analysis. Table 3 and Table 4 show the results obtained when the algorithms proposed in this chapter are compared with comparative models for defect segmentation. The best results are obtained on the self-constructed dataset with an average Pi.AUROC result of 96.6%, which is 3.2% higher than that of PFM, while the Pi.AUROC of this method is as high as 98.8% for the broken type defects, which is much better than that of other models. Under the F1-score evaluation index, this paper’s method achieves 97.2%, 93.4%, and 94.6 on the three types of defects compared to ET-STPM, which is 2.5%, 3.3%, and 2.1% higher, respectively. In addition, in order to comprehensively assess the performance of this paper’s method in segmenting insulator surface defects, this section further visualizes the heatmap visualization the comparison model, as shown in Figure 9. In the first and second rows, for the defects of breakage and self-explosion types, Padim and UniAD detect the defects’ approximate locations as well, but their defect localization is not precise enough and their defects’ heatmap intensity are more than the real anomaly area compared to GT, while this paper’s method demonstrates the accurate defect localization capability at pixel level, and the defect boundaries and coverage areas are more in line with the reality. In the third line of flash defect detection, this method also shows better defect detection ability.

#### 4.4.2. Comparison Experiment on CID Dataset

**Figure 10:**
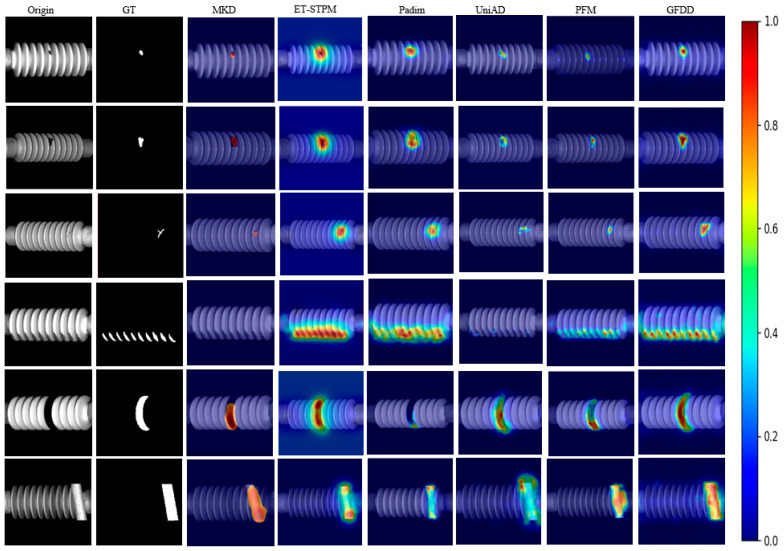
Results of CID heat map visualization.

To further validate the effectiveness of this paper’s method in the field of insulator surface defect detection, this section uses the publicly available dataset CID for comparative experimental analysis, which covers six types of defects, namely breakage, contamination, crack, dirt, missing, and shelter. as can be seen from Table 5, Table 6 and Table 7 with the current mainstream network, this paper’s method improves 1.2% on Im.AUROC compared to PFM model, and improves 1.9% on Pi.AUROC compared to other teachers’ network model ET-STPM model, and on F1 metrics, this paper’s method achieves the optimal results on multiple defect types. Also combined with the visualization, compared to the Padim model, this paper’s method is more accurate in pixel-level detection and has fewer missed detections. Compared with the ET-STPM model, it is more consistent with the mask in detecting defects at different scales, and it has fewer pixel-level misdetections, and the boundaries of defects are more consistent with the shape of the mask. In order to demonstrate the superiority of the proposed method more intuitively, heatmap visualization is performed in this section, as shown in Figure 10. In the first row, for broken defects, Padim and ET-STPM cannot accurately locate the defects and produce a large number of mis-detected regions, while the PFM method can accurately locate the defects but the defect boundaries do not match well with the actual masks, compared to which the method in this paper achieves a more accurate defect localization; In the fourth and fifth lines, in the face of irregular defects, the heat map of the defects in the MKD and UniAD occurs with different degrees of leakage detection, while the defect heat map of this paper’s method is almost identical to the actual defect shape and location, demonstrating a more comprehensive detection capability.

#### 4.4.3. Comprehensive Performance Comparison Analysis

In practical industrial application scenarios, although pixel-level detection accuracy is an important indicator of the model, the performance of model inference speed, computational complexity, and stability should not be neglected in practical deployment as well. Therefore, this subsection discusses and analyzes the above metrics on a self-built dataset using the experimental environment of Section 4.2 to highlight that the proposed method has superior comprehensive performance, and the specific results are shown in Table 8.

**Figure 11:**
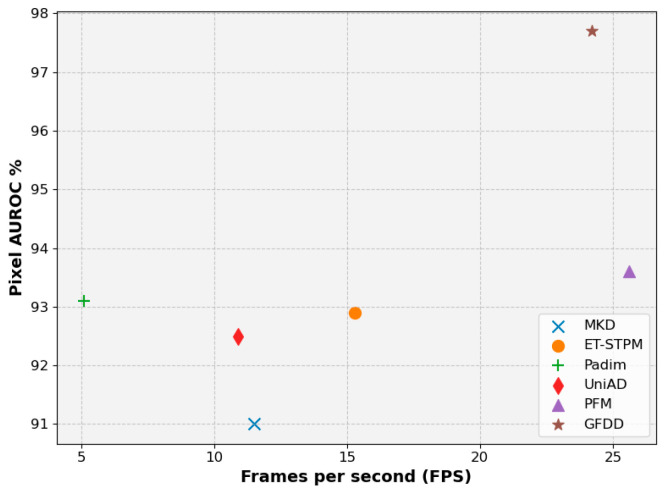
Comprehensive comparison of detection speed and detection accuracy.

Through Table 8, we can find that in terms of the number of model parameters, Padim model achieves the best results in terms of the number of parameters because it only uses the pre-trained network as the backbone, but in terms of the computational complexity, it is high due to the fact that Padim uses a multivariate Gaussian distribution modeling for each patch and calculates the anomaly scores for each pixel point by using the Mahalanobis distance. slower detection rate. Compared with MKD and ET-STPM, GFDD has a slight increase in the number of parameters due to its dual-teacher structure, but the method in this paper achieves better results in terms of computational efficiency and running time because it uses diffusion distillation to avoid the direct and complex operation of multi-level features. Compared with the PFM method, the method in this section is slightly inferior in terms of computational efficiency, but in combination with Figure 11, GFDD has a more significant advantage in pixel-level detection accuracy, and has the optimal overall performance in real deployment scenarios.

In addition to further validate the convergence speed and stability of the model, this subsection provides a more comprehensive analysis of the methodology of this paper by comparing the pixel-level accuracy curves as well as the confusion matrices during the training of different models.

**Figure 12:**
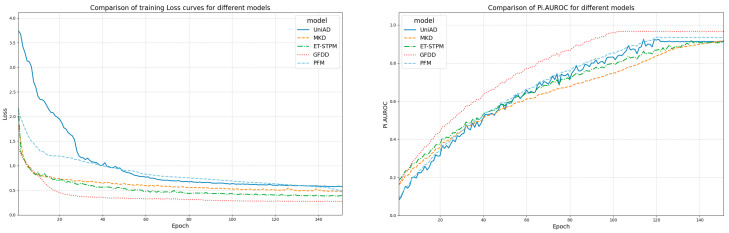
Compare the loss of different models and the Pi.AUROC.

The visualization in Figure 12 shows that GFDD exhibits a more stable convergence speed during the training process compared to the existing teacher-student networks ET-STPM and MKD. In terms of pixel-level accuracy, the use of graph feature consistency constraints mitigates the feature bias before training, making the model accuracy fluctuate less during the ascent process, and it is easier to get out of the local optimal solution. At the same time, the diffusion distillation method adopted by GFDD helps the network achieve better pixel-level accuracy, which is more in line with the needs of practical application scenarios.

**Figure 13:**
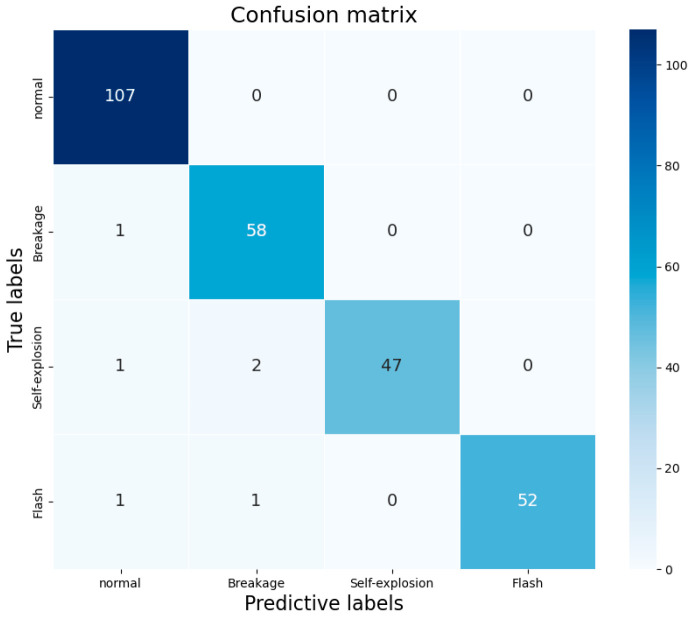
Confusion matrix analysis.

Finally, through the analysis of Figure 13, it can be found that the diagonal region is found to be dark and the non-diagonal region is found to be light, which intuitively reflects that the number of correct classifications is much more than the number of incorrect classifications, and that the overall performance of the model is better, but there is a misidentification of the type of defects in the differentiation of part of the defects, and it is analyzed that it may be that part of the flashback defects and the breakage defects have a certain degree of feature similarity, which leads to misdetection, and it is reflected from the side that the model’s bottom layer feature extraction ability needs to be further strengthened.

### 4.5. Ablation Experiment

In order to deeply investigate the impact of each component on the anomaly detection results, a series of ablation experiments are designed in this section to validate the effectiveness of its modules. The relevant parameters used in the experiments of this section are consistent with Section 4.2.

#### 4.5.1. Comparative Validity Analysis of Graph Features

**Figure 14:**
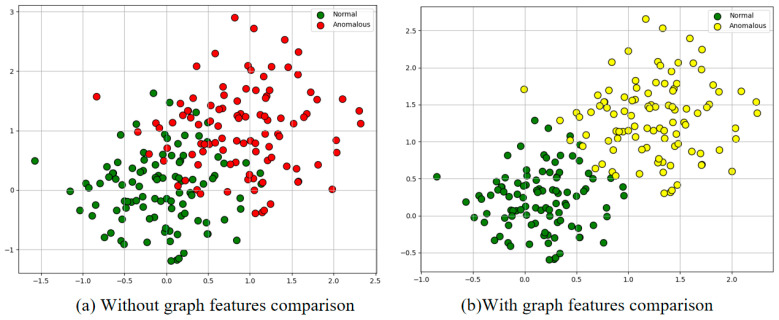
T-SNE visualization comparison. (**a**) Adopting graph feature consistency comparisons. (**b**) Unadopted graph feature consistency comparisons.

In this section, the validity of graph feature comparison is verified by setting up two sets of experiments. the first set of experiments adopts a two-teacher structure, with Teacher 1 using pre-trained WideResNet50 and Teacher 2 using unpretrained WideResNet50. the second set of experiments employs the same structure as the first set of experiments, with the only variable being the second set of experiments employing graph feature comparison. Table 9 presents in detail the results of the quantitative comparison of the two sets of experiments. Through the comparative analysis, it can be found that after the introduction of graph feature comparison, Im.AUROC, Pi.AUROC, and F1-score are improved by 2.6%, 2.9%, and 1.7%, respectively, compared with the first group.Meanwhile, this paper analyzes the last layer of encoder features of the Teacher’s Network 2 by using the T-SNE [39], and the results of which are shown in, after employing the graph feature consistency comparison. The results are shown in Figure 14, where we analyze the feature distribution of the target domain through t-SNE visualization: (a) When graph feature consistency is not used (left panel), the positive samples are represented in green and the anomalous samples are represented in red, and there is an obvious overlap between them in the feature space, which indicates that the domain bias leads to the degradation of the feature discriminative performance; (b) After using the graph feature consistency comparison method in this paper (right figure), the feature clustering of positive samples and anomalous samples is improved and the distinction between feature boundaries is more obvious, which proves that the method effectively alleviates the problem of domain bias by aligning feature distributions through graph feature consistency.

#### 4.5.2. Comparison of Teachers’ Network Backbones

In this section, the experimental analysis is conducted on the self-constructed dataset by replacing different backbone networks as the teacher network, as shown in Table 10, which presents the quantitative comparison results of different network models as the backbone network of the teacher network in detail. The comparative analysis reveals that the deep network structure shows better performance on the anomaly detection task, comparing with the shallow ResNet18, the Im.AUROC, Pi.AUROC, and F1-score are improved by 6.3%, 4.9%, and 6.5%, respectively, when using WideResNet5. The analysis may be that its multi-scale feature fusion mechanism can better extract sample features with stronger feature characterization ability. Based on the above comparative analysis results, this paper finally selects the WideResNet50 network as the backbone network in the teacher network in order to give full play to its advantages in defect detection and to improve the performance of the model.

#### 4.5.3. Modular Ablation Experiments

In this section, using the traditional teacher-student network [27] as a base model, systematic ablation experiments are conducted for the graph feature comparison mode, the interlayer feature fusion mode, and the channel attention mode, and the components are introduced step by step to analyze their effects on the insulator defect detection performance. As shown in Table 11, the base model is 88.6% and 89.9% in the test set Im.AUROC and Pi.AUROC, respectively. And after the introduction of GFCM module alone, Im.AUROC and Pi.AUROC are improved by 4.9% and 4.3%, respectively, indicating that the two-teacher map feature consistency comparison approach significantly mitigates the feature distribution bias problem; further superimposed with the IFFM module, which enhances the multi-scale defects characterization through cross-layer feature interactions, Im.AUROC and Pi.AUROC reaches 95.2. 4% and 75.8%; finally, after introducing the CAFM module, the model Im.AUROC and Pi.AUROC reach the highest values of 97.7% and 96.6% by means of diffusion distillation from deep to shallow. The experiment proves that the proposed modules jointly construct a hierarchical feature optimization mechanism, which significantly improves its comprehensive performance in defect detection.

### 4.6. Generalization Experiment

**Figure 15:**
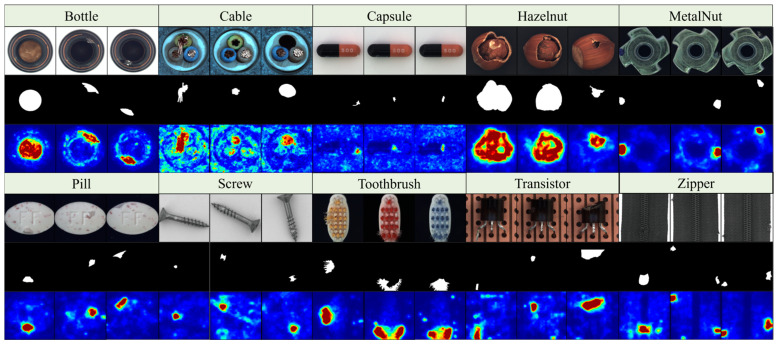
MVtec AD heat map visualization results.

To further validate the generalization of this paper’s method, experimental validation is carried out in this section on the public industrial surface defect dataset MVtec AD, which is visualized as shown in Figure 15. The proposed method is not only effective in locating the defect location on small defects such as Screw and Pill, but also for irregular defects on complex images such as Cable, Toothbrush and Hazelnut, the thermally visualized area is highly consistent with the actual defect area, which further proves that this paper’s method has good robustness. The quantitative analysis in Appendix A also validates the excellent performance of this method in the task of surface defects in general industrial products.

## 5. Discussion

### 5.1. Research Limitations

Although the insulator surface defect detection method based on graph feature diffusion distillation proposed in this study significantly improves the pixel-level accuracy of insulator defect detection, there are still some limitations that need to be improved. First, the diversity of the dataset may not be sufficient to cover all real-world scenarios (e.g., in low-light environments with complex backgrounds), which may affect the generalization ability of the model. Second, the robustness and accuracy of the model at low resolution have not been fully validated. In addition, the model relies on some hardware support compared to existing methods, and the limitation of computational resources may affect the deployment and inference speed.

### 5.2. Future Work

In order to improve the performance and application scope of the methods in this paper, future research can focus on several key directions. First, although the current model performs well on multiple datasets, it needs to be validated on a wider and more diverse range of datasets, especially when dealing with complex real-world environments. In addition, future research could extend this model to other power equipment detection tasks or joint detection of multiple tasks using multimodal data. At the same time, considering the constraints of computational resources, more lightweight network architectures will be further investigated in the future.

## 6. Conclusions

To reduce dependence on labeled data, achieve pixel-level defect detection on insulator surfaces, and improve detection accuracy for unknown defects, this paper proposes a defect detection algorithm based on graph feature knowledge refinement. Our approach employs a dual-teacher structure with graph feature consistency comparison to enhance feature uniformity across normal samples, thereby mitigating feature bias inherent in pre-trained networks. An inter-layer feature fusion module delivers high-quality input features to the student network, while a novel channel attention module with diffusion distillation progressively guides multi-layer feature learning from deep to shallow levels. Experimental validation confirms the method’s effectiveness in detecting defects on insulator surfaces and industrial products with accurate pixel-level localization. Although demonstrating strong comprehensive performance, practical deployment requires further optimization for challenging scenarios involving occlusion and low illumination contrast. Subsequent phases will incorporate text and thermal imaging data through multimodal fusion to enhance unsupervised defect detection capabilities, concurrently pursuing lightweight network architectures to reduce computational resource demands.

## Figures and Tables

**Table 1:** Wins dataset details.

Defect Type	Training Set	Test Set (Normal)	Test Set (Abnormal)
breakage	389	38	59
self-explosion	345	32	50
flash	368	37	54

**Table 2:** Different methods on Wins dataset Im.AUROC (%).

Method	Average	Breakage	Self-Explosion	Flash
MKD	91.0	89.1	91.3	92.6
ET-STPM	92.9	90.6	93.8	94.4
Padim	93.1	93.9	94.9	90.5
UniAD	92.5	95.7	90.6	91.3
PFM	93.6	92.9	86.6	95.4
GFDD	**97.7**	**98.2**	**96.4**	**98.5**

**Table 3:** Different methods on Wins dataset Pi.AUROC (%).

Method	Average	Breakage	Self-Explosion	Flash
MKD	90.7	92.6	85.6	94.0
ET-STPM	91.8	91.3	93.8	90.4
Padim	92.3	95.7	92.2	88.9
UniAD	91.3	92.8	90.7	90.3
PFM	93.4	96.5	91.2	92.4
GFDD	**96.6**	**98.8**	**95.2**	**95.8**

**Table 4:** Different methods on Wins dataset $F_{1}$ (%).

Method	Average	Breakage	Self-Explosion	Flash
MKD	90.9	93.2	89.9	89.5
ET-STPM	92.4	94.7	90.1	92.5
Padim	91.0	93.8	91.2	87.9
UniAD	92.7	94.5	91.4	92.1
PFM	90.8	91.4	88.3	92.6
GFDD	**95.1**	**97.2**	**93.4**	**94.6**

**Table 5:** Different methods on CID dataset Im.AUROC (%).

Method	Average	Breakage	Contamination	Crack	Dirt	Missing	Shelter
MKD	92.7	93.3	92.4	94.1	94.6	92.4	89.6
ET-STPM	92.9	92.1	94.3	92.9	93.7	93.5	91.0
Padim	93.2	94.3	93.9	93.5	95.2	91.2	91.3
UniAD	93.9	94.7	93.2	95.9	94.8	94.7	89.9
PFM	94.0	95.4	**95.7**	94.3	94.1	93.2	91.2
GFDD	**95.2**	**96.6**	**95.7**	**96.2**	**95.1**	**94.9**	**92.6**

**Table 6:** Different methods on CID dataset Pi.AUROC (%).

Method	Average	Breakage	Contamination	Crack	Dirt	Missing	Shelter
MKD	93.1	93.4	93.6	93.2	92.4	92.1	93.9
ET-STPM	92.6	92.5	91.7	92.7	92.7	92.1	93.7
Padim	93.1	94.2	92.9	93.1	93.2	92.7	93.5
UniAD	93.4	93.9	93.9	93.5	92.9	91.9	94.0
PFM	93.6	95.4	94.1	92.4	93.6	92.7	93.2
GFDD	**94.5**	**96.7**	**95.4**	**93.8**	**93.7**	**93.1**	**94.2**

**Table 7:** Different methods on CID dataset $F_{1}$ (%).

Method	Average	Breakage	Contamination	Crack	Dirt	Missing	Shelter
MKD	89.5	89.2	89.5	91.6	91.4	91.7	83.3
ET-STPM	89.6	90.1	90.2	90.3	92.6	89.9	84.7
Padim	90.3	90.7	91.3	92.0	91.7	90.3	85.8
UniAD	91.8	91.6	91.1	92.9	92.0	91.1	91.9
PFM	92.0	90.9	92.5	**93.1**	92.1	91.5	91.8
GFDD	**93.7**	**92.2**	**93.1**	**93.1**	**92.4**	**98.9**	**92.3**

**Table 8:** Computational efficiency comparison results.

Method	Param(M)	Flops(G)	FPS
MKD	34.7	40.6	11.5
ET-STPM	32.8	37.2	15.3
Padim	**11.7**	45.6	5.1
UniAD	48.4	50.7	10.9
PFM	26.8	**31.2**	**25.6**
GFDD	35.5	32.6	24.2

**Table 9:** Ablation analysis results.

Model ID	Im.AUROC	Pi.AUROC	F1
I	91.1	91.3	91.0
II	**93.7**	**94.2**	**92.7**

**Table 10:** Backbone network ablation comparison results.

Model ID	Im.AUROC	Pi.AUROC	F1
ResNet18	91.4	91.7	88.6
ResNet34	93.7	92.1	91.0
ResNet50	95.2	94.8	92.7
WideResNet50	**97.7**	**96.6**	**95.1**

**Table 11:** Modular Ablation Results.

Base	GFCM	IFFM	CAFM	Im.AUROC	Pi.AUROC	F1
√				88.6	89.9	87.7
√	√			93.7	94.2	92.7
√	√	√		95.2	94.9	94.3
√	√	√	√	**97.7**	**96.6**	**95.1**

## Data Availability

Due to privacy reasons, all data supporting this study cannot be made fully public and more details can be obtained from the corresponding author upon request.

## Appendix A

Quantitative analysis results of this paper’s method with other existing methods on the mvtec dataset.

**Table A1:** Im.AUROC of different methods for object categories on the MVTec AD dataset (%).

Method	Average	Bottle	Cable	Capsule	Hazel Nut	Metal Nut	Pill	Screw	Tooth Brush	Transis Tor	Zipper
MKD	85.3	99.4	89.2	80.5	73.6	73.6	82.7	83.3	92.2	85.6	93.2
ET-STPM	91.7	98.9	93.2	91.2	90.3	92.6	89.9	84.7	93.1	92.4	91.5
Padim	93.7	99.9	92.7	91.3	92.0	98.7	93.3	85.8	**96.1**	97.4	90.3
UniAD	95.3	**100**	97.6	85.3	99.9	99.0	88.3	91.9	95.0	**100**	96.7
PFM	96.5	**100**	98.8	94.5	**100**	**100**	96.5	91.8	88.6	97.8	**97.4**
Ours	**97.5**	99.7	**99.2**	**95.2**	99.9	**100**	**98.9**	**92.3**	94.5	99.9	95.9

**Table A2:** Pi.AUROC of different methods for object categories on the MVTec AD dataset (%).

Method	Average	Bottle	Cable	Capsule	Hazel Nut	Metal Nut	Pill	Screw	Tooth Brush	Transis Tor	Zipper
MKD	90.7	96.3	82.4	95.9	94.6	86.4	89.6	96.0	96.1	76.5	93.9
ET-STPM	97.5	98.1	94.9	**98.9**	99.0	**98.5**	**98.0**	98.5	98.0	86.9	**99.7**
Padim	97.7	98.3	96.7	98.5	98.2	97.2	95.7	98.5	98.8	97.5	98.5
UniAD	97.1	98.1	**96.8**	97.9	98.8	95.7	95.1	97.4	97.8	98.7	96.0
PFM	96.9	98.4	96.7	98.3	**99.1**	97.2	97.2	98.7	98.6	87.8	98.2
Ours	**98.2**	**98.6**	95.7	98.5	**99.1**	97.1	97.9	**99.0**	**98.7**	**98.5**	99.0
